# Structural Characterization and Adsorption Properties of Dunino Raw Halloysite Mineral for Dye Removal from Water

**DOI:** 10.3390/ma14133676

**Published:** 2021-07-01

**Authors:** Simona Filice, Corrado Bongiorno, Sebania Libertino, Giuseppe Compagnini, Leon Gradon, Daniela Iannazzo, Antonino La Magna, Silvia Scalese

**Affiliations:** 1 Istituto per la Microelettronica e Microsistemi, Consiglio Nazionale delle Ricerche (CNR-IMM), Ottava Strada n.5, I-95121 Catania, Italy; simona.filice@imm.cnr.it (S.F.); corrado.bongiorno@imm.cnr.it (C.B.); sebania.libertino@imm.cnr.it (S.L.); antonino.lamagna@imm.cnr.it (A.L.M.); 2 Dipartimento di Scienze Chimiche, Università di Catania, Viale A. Doria 6, 95125 Catania, Italy; gcompagnini@unict.it; 3 Faculty of Chemical and Process Engineering, Warsaw University of Technology, ul. Warynskiego 1, 00-645 Warsaw, Poland; Leon.Gradon@pw.edu.pl; 4 Dipartimento di Ingegneria, Università degli Studi di Messina, Contrada di Dio, I-98166 Messina, Italy; diannazzo@unime.it

**Keywords:** halloysite, clay, water purification, dyes removal, adsorption

## Abstract

In this work, raw halloysite mineral from Dunino (Poland) has been characterized and tested as an efficient and low-cost adsorbent for dye removal from water. The morphology and structure of this clay were characterized using scanning electron microscopy (SEM) and transmission electron microscopy (TEM) and the chemical composition was evaluated by means of X-ray fluorescence spectroscopy (XRF), energy dispersive X-ray spectroscopy (EDX), and electron energy loss spectroscopy (EELS). The results showed that it is made up of both platy and tubular structures, mainly composed of Si, Al, and O. Iron oxide particles covering the platy structures were also observed. The surface charge of halloysite was measured by z-potential measurements and by the evaluation of the point of zero charge. The clay was tested as an adsorbent for the removal of positively and negatively charged dye molecules, i.e., methylene blue (MB) and methyl orange (MO), both separately and in a mixed-dye solution. Halloysite showed the ability to efficiently and selectively remove MB molecules by adsorption, both in a single-dye solution and in a mixed one. The adsorption of positive dyes on the clay surface mainly occurred through ion exchange at negatively charged sites on its surface. The possibility of regenerating the clay for further dye removal processes is also shown.

## 1. Introduction

Despite their detrimental effects on the environment and prohibitive agreements [1], more than 100 highly toxic and potentially carcinogenic dyes are still available on the market. They represent highly dangerous pollutants, since around 15–50% of azo-type textile dyes are released into wastewater during the dyeing process. This water is commonly used, in developing countries, for the purpose of irrigation in agriculture [2,3], despite the fact that it is very damaging to soil microbial communities and to the germination and growth of plants. The diseases induced by textile dyes on humans can include dermatitis, disorders of the central nervous system [4], or to the inactivation of enzymatic activities themselves by the substitution of enzymatic cofactors [5]. It is, therefore, essential to use treatment strategies for dye removal through physical, chemical, and biological technologies, or combinations thereof [6]. In this regard, adsorption techniques are widely used due to their simplicity, high efficiency, easy recovery, and reusability of the adsorbent [7], and they provide an attractive alternative treatment, especially if the adsorbent is inexpensive and readily available [8].

Activated carbon has shown high adsorption capacity for the removal of various pollutants. However, it has relatively high operational costs, mainly due to its difficult recovery from water and regeneration after use [8]. As an alternative, carbon nanomaterials, such as graphene oxide, have been tested for the removal of water contaminants, both as adsorbent and in photocatalytic processes. The latter represent a promising methodology to achieve complete organic pollutant degradation, or to transform them in harmless species. In this regard, inorganic oxides, such as titanium dioxide (TiO_2_) and bismuth oxide (Bi_2_O_3_), have been investigated for dye degradation purposes and water disinfection [9,10,11] by directly dispersing them in water [12,13], in combination with carbon nanostructures [14,15], or as active fillers by embedding them in polymeric films [16,17,18]. Good results in dye degradation were obtained, along with the possibility of easy recovery of the photocatalyst as well as its reuse. However, the high production costs of graphene oxide and inorganic semiconductors limit their use in practical water treatment.

Recently, dispersed minerals, such as, kaolinite, have been investigated as low-cost adsorbents for water pollutants; these materials do not require complex preparation and their adsorption capacities are sufficiently high to be considered as efficient and low cost alternatives [11,19,20] to previously listed ones. Halloysite belongs to the kaolin group of minerals, and consists of stacked pairs of tetrahedral silica sheets and octahedral alumina sheets [21,22].

Negatively charged sites in the tetrahedral sheets originate from the replacement of Si(IV) atoms by Al(III) atoms and terminal hydroxyl moieties are present at the edges. Depending on the pH, these groups can be protonated or not, affecting their charge. Because of these features, halloysite could adsorb ions and neutral molecules (i) through ion exchange at negatively charged sites in the tetrahedral sheets or (ii) by the formation of surface complexes with both the Al–OH and Si–OH groups located at the layer edges. The adsorption properties of this clay depend on its chemical composition, crystalline structure, and the purification procedures employed. Halloysite has a considerably large specific surface area and a high ion exchange capacity. It is also characterized by good chemical and thermal resistance at low and moderate temperatures [23,24]. Iron oxides or oxyhydroxides can be present in such clays, conferring a typical brown color to the mineral [23,24].

Kaolinite and tubular halloysite were studied as efficient adsorbents for the removal of cationic and anionic aqueous dyes which are naturally present or after calcination or treatment with acid and basic medium [25,26,27,28,29,30]. The removal efficiency of halloysite is higher than most of conventional adsorbents, and it has been tested as novel nanomaterial that can be used in water filter system [25]. Harrou et al. showed that a raw kaolinite and halloysite could be successfully utilized for the adsorption of methylene blue (MB) dye from aqueous solutions [26]. In particular, raw halloysite has a higher adsorption capacity of MB than kaolinite. Similarly, Luo et al. chemically modified raw HNTs by treatment with HCl followed by NaCl [27]. This treatment leads to a low-cost adsorbent with a good affinity for positively charged species with respect to bare HNTs. The adsorption mechanism of MB onto C-HNTs was explained by ion exchange of the MB cation with sodium ion or protons generated from the adsorbent surface. Commercial kaolin from a Polish deposit has been examined as a low-cost adsorbent for effluent remediation using five cationic and five anionic industrial dyes [30]. This clay was shown to be a rapid and effective adsorbent for cationic dyes, whereas the anionic dyes exhibited no affinity to the kaolin surface.

An example of anionic dye is MO, a synthetic azo dye, generally used as a coloring agent in textile and leather industries, in printing, paper manufacturing, pharmaceutical, and in food processing industries [31]. MO is a carcinogenic water-soluble azo dye, and is also acknowledged as an acidic or anionic dye. It can cause vomiting and diarrhea. High levels of exposure to MO can result in death. Furthermore, by-products generated from its degradation or metabolization are toxic. Among cationic dyes, MB is one of the most studied and is a basic dye that is usually applied in textiles, plastics and in the pharmaceutical industry [32]. During degradation it releases aromatic amines, being a potential carcinogen [33]. The water contamination with MB at higher concentrations may cause health problems, such as allergic reactions, vomiting, skin problems, respiratory track diseases, heart problems, and neurotoxicity, cytotoxicity, and mutagenicity effects.

The work described herein concerns a detailed microstructural characterization of raw halloysite mineral from Dunino mine and its use for the adsorption of MB and MO dyes from water. For these raw materials without any purification or activation treatment, we report adsorption efficiencies better or comparable with results achieved in the literature by similar materials (after purification treatments) [26,27,28,29,30]. Furthermore, the adsorption selectivity of raw halloysite was also investigated, using both single and mixed-dye (MB + MO) solutions. The latter experiment on the adsorption selectivity of raw halloysite shows that the mineral is selective towards the adsorption of MB with respect to MO, and, for the first time to our knowledge, the MB adsorption rate is shown to be enhanced in mixed-dye solutions. Furthermore, the possibility of regenerating halloysite after dye adsorption and its reuse for new adsorption processes with similar performance are also shown.

Possible applications in industrial wastewaters treatments could take advantage of the low cost and adsorption properties of such clay powders.

## 2. Materials and Methods

Raw halloysite mineral was obtained from the Dunino mine (Poland) [23]. The Dunino deposit is located in Lower Silesia (this is one of the currently exploited deposits of this mineral in the world) containing at least 10 million tons of homogeneous raw materials, and is an open pit mine. Clay extracted from this deposit is a product of basalt weathering. Before each experiment, the clay powder was weighed and dispersed in a suitable water volume using ultrasonic treatment for 3 h in order to obtain the desired clay concentration.

Morphology and chemical mapping of the samples was performed using a field emission scanning electron microscope (Supra 35 FE-SEM by Zeiss, Oberkochen, Germany) equipped with an energy dispersive X-ray (EDX) microanalysis system (X-MAX, 80 mm^2^ by Oxford Instruments, Abingdon, UK). The clay powder was dispersed in water and some drops of the dispersion were deposited on a polymeric film which was used as a substrate.

Transmission electron microscopy (TEM) was used for the nanoscale structural characterization. To make the specimen suitable for TEM observations, an aqueous dispersion containing the clay powder was dropped on a lacey-carbon TEM grid. TEM analyses were performed using a Jeol JEM-2010F at 200 keV. Decreased beam intensity was used during TEM analysis in order to avoid sample modification; nevertheless, we noticed that some tubular structures were quickly modified under the electron beam. Scanning TEM and EELS investigations were instead performed with a JEOL ARM200F in the scanning configuration.

The surface charge of halloysite powder was measured using a Horiba Scientific NanoParticle Analyzer SZ-100-Z in water (pH = 5.8) and in presence of methyl orange (10^−5^ M), methylene blue (10^−5^ M), and a mixture of both the dyes at the same concentration.

The point of zero charge (PZC) of the clay dispersion was measured according to [34]. First, the pH of different aliquots (5 mL each) of 0.1 M NaCl solution were adjusted in the range 2–10, using either dilute HCl or NaOH aqueous solutions. After that, 0.5 mg of halloysite was added to each glass vial. The mixtures were then agitated at room temperature to attain equilibrium. The final pH values of each dispersion were recorded and the pH change was plotted versus the initial pH value in order to determine the pH_PZC_ of the clay.

The elemental composition of the halloysite material was performed using a Rigaku Supermini200 (Tokyo, Japan), a benchtop wavelength dispersive X-ray fluorescence (WDXRF) spectrometer equipped with a Pd X-ray tube for X-ray production. The sample was included in a pressed pellet using boric acid as binder; the concentrations of the major and minor elements were calculated using ZSX software.

The adsorption ability of this clay was investigated for the adsorption of MB and MO. Clay powder was directly dispersed in 5 mL of MB and MO solutions (10^−5^ M), respectively, at different concentrations (0.115 and 0.23 mg/mL). The clay concentration values were suitably chosen in order to keep the suspensions stable and avoid precipitation. The dye molecule removal efficiency was evaluated by measuring changes in the UV-Vis absorbance spectra acquired using a UV-Vis spectrophotometer (Cary^®^ 50 UV/vis by Agilent Technologies, Santa Clara, CA, USA), in a wavelength range between 200 and 800 nm, using wide optical window quartz cuvettes (200–2500 nm). In particular, MO and MB dye concentrations were evaluated by means of the Lambert–Beer law, considering the absorbance peaks at 465 nm and 664 nm for MO and MB, respectively. The selectivity of this clay was evaluated by comparing its adsorption ability for MO and MB in a mixed solution (1:1, here named “Green”) composed by MO and MB at 10^−5^ M, respectively.

Further adsorption experiments were performed by maintaining the halloysite concentration at 0.23 mg/mL and varying the initial MB concentration (3.2, 6.4, 9.6, and 12.8 mg/L). UV-Vis spectra were acquired after equilibrium was reached. The data were fitted with Freundlich and Langmuir isotherms in the single-dye or mixed-dye solutions. In the last case, the MO concentration was also increased (3.3, 6.6, 9.9, and 13.2 mg/L, respectively) keeping the MB:MO molar ratio unaltered (i.e., 1:1). For each experiment, the dye concentration (C_e_ mg/L) and the amount of adsorbed dye for unit mass of adsorbent (Q_e_ mg/g) at equilibrium were determined. Equilibrium was reached after some hours of contact between the clay and the dye for low MB concentrations and up to 2 days for mixed-dye solutions at higher concentrations. The Langmuir and Freundlich constants with Q_max_ and 1/*n* values were obtained from the plots of the linearized equations, as reported in Section 3.3.3.

Regeneration of halloysite was performed after MB adsorption, in order to use it again for further MB removal treatments. The clay was recovered from water after MB removal (3-h process) and when it was dry it looked blue. It was heated on a hot plate at 200 °C in air until it turned back to its original color (time >4 h). The adsorption ability of the regenerated halloysite was tested according the same procedure used for the original clay, as described above.

## 3. Results and Discussion

### 3.1. Morphological and Chemical Characterizations

**Figure 1:**
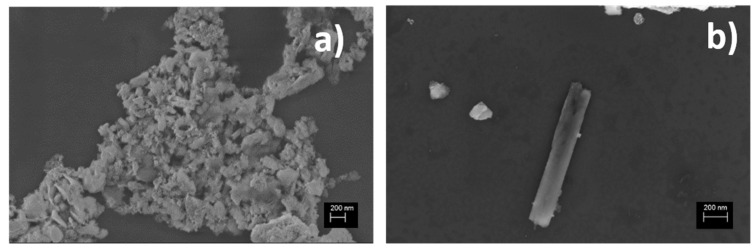
SEM images acquired on platy (**a**) and rolled (**b**) structures of raw halloysite mineral.

Figure 1 shows SEM images at different magnifications of raw halloysite mineral powder, deposited on a polymeric substrate for analysis purposes, showing platy and tubular structures, typical of material from the Dunino deposit. The surfaces of the flakes were irregular, heterogeneous, and porous. The tubes were formed by layer rolling caused by the dimensional misfit between octahedral and tetrahedral sheets and weak interlayer bonding [35]. The length distribution of halloysite tubules observed in the literature covers a wide range, from 0.02 to 30 μm, whereas their widths range from 0.05 to 0.2 μm. In Figure 1b, we show a SEM image of a typical tube observed in our material with a length of about 1 μm and a width of about 200 nm. The greater curvature of the core of halloysite crystals seems to be linked to a smaller number of stacked layers [36].

In order to investigate the chemical composition of the material, we performed EDX analyses during SEM observation. As an example, Table S1 of the Supplementary Information reports the wt% of elements measured by EDX analysis in two different areas of the sample shown in Figure S1 of Supplementary Information. The flakes and the tubes were made up of silica and alumina layers. The roughness was due to the presence of iron oxide nanoparticles on the flake surfaces, while lower or negligible amounts of iron oxide particles were observed on the tubular structures.

The mass-averaged quantitative and qualitative EDX elemental analyses confirmed that the most abundant elements were oxygen, aluminum, silicon, and iron, while the carbon signal came from the polymeric substrate used during analyses. Silicon and aluminum were equally present, and their relative Si/Al ratio was about 1 (0.8 < Si/Al < 1.5). Iron was distributed throughout the whole sample at variable percentages: the Fe/Si ratio was lower than the Fe/Al one and the relative iron amount was found to be higher in the thicker and rough layers. In tubular structures, the Si/Al ratio is about 1, similarly to platy ones, but the amount of iron is lower than 1%. In addition to iron, other impurities, such as Ti and Mg, were observed by XRF analysis, shown below, and also reported in [31]. Al and Fe are found as substitutional impurities for Si in the tetrahedra and Fe, Mg, and Ti for Al in the octahedra, causing local imbalances of electric charges and creating a number of so-called active sites able to form bonds with many different substances [37].

As a further confirmation of clay chemical composition, the halloysite material powder was dispersed in boric acid and XRF spectra were acquired. The chemical composition of the halloysite measured by XRF spectroscopy is reported in Table 1.

The results obtained by XRF indicate that the Si/Al ratio was 1.13, the Fe/Si ratio was 0.76, slightly lower than the 0.85 obtained for the Fe/Al ratio. Elemental analysis performed on the raw halloysite mineral showed results similar to those reported in the literature [38]; however, looking at the microscopic structures from the SEM/EDX analysis, the chemical compositions can be locally different in terms of the content of Fe and other impurities, because of the volcanic origin of this mineral.

**Figure 2:**
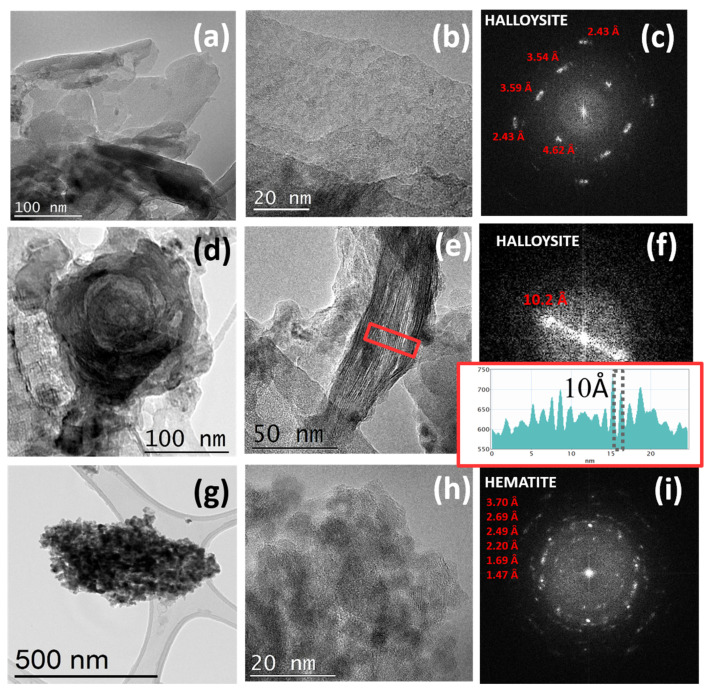
TEM images at different magnifications of planar (**a**,**b**) and rolled (**d**,**e**) aluminosilicate layers and iron oxides agglomerates (**g**,**h**). For each structure, the FFT patterns are shown (**c**,**f**,**i**).

We performed TEM characterization combined with EELS spectroscopy to shed light on the structural and local chemical composition of halloysite from Dunino. Figure 2 reports TEM images of different structures present in the halloysite material at different magnifications and the corresponding fast Fourier transformation (FFT) patterns extracted from the high-resolution images in Figure 2b,e,h.

The two different configurations of aluminosilicate layers observed, i.e., platy and rolled, are reported in Figure 2a,b,d,e, respectively. As reported in the Introduction, Dunino clay consists of tetrahedral Si_2_O_5_ sheets and octahedral Al sheets, respectively [39]. A scheme of its structure is reported in Figure S2 of the Supplementary Information. Silicon is located at the center of the tetrahedron, and oxygen anions form the four corners. The individual tetrahedron shares three corners (the three basal oxygens) with adjacent tetrahedra, constituting a hexagonal mesh arrangement and the apical oxygen forms part of the octahedral sheet. The octahedral sheets comprise medium-sized cations at the octahedron center (usually Al, Fe^2+^, or Fe^3+^), and oxygens at the eight corners. The individual octahedral units are laterally linked with hydroxyls. The OH groups are located at the center of each of the tetrahedral six-fold rings (hexagonal arrangement), at the same level as the apical oxygens. The layer repetition defines the (001) basal spacing of the unit cell; this spacing is characteristic of the type of stacking present. Two different structures with different interlayers distances, i.e., 10 Å and 7 Å, are generally reported according to the higher or lower hydration states, respectively [39]. The FFT pattern extracted by the high-resolution image in Figure 2b can be very well fitted by halloysite crystal (both 10 Å and 7 Å) as long as the b axis of the cell is increased to 9.25 Å (see Figure 2c), far from the tabulated 8.9 Å value reported for the pure material. This large difference in the b parameter could be ascribed to the presence of iron atoms within the crystal and, in particular, to the partial isomorphous substitution of Fe^3+^ for Al^3+^ in the octahedral sheet [40].

**Figure 3:**
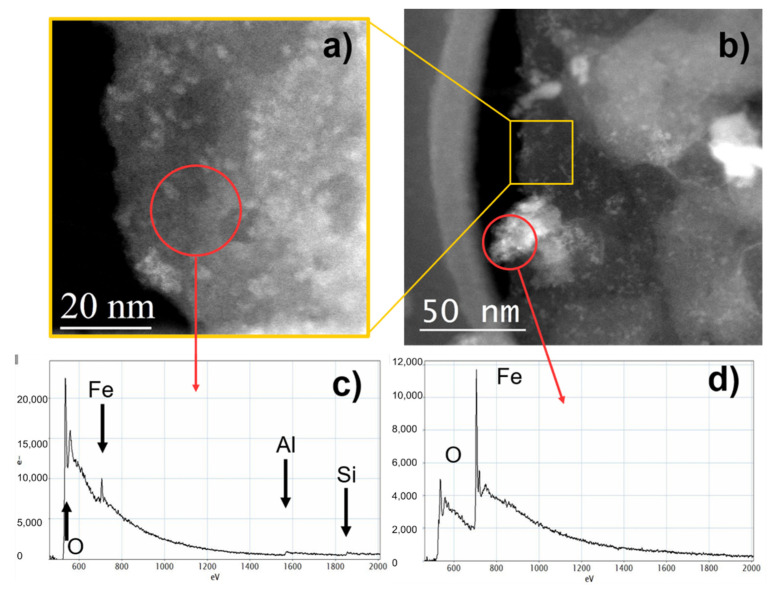
STEM images of platy layers at high (**a**) and low (**b**) magnifications. EELS spectra of platy structure (**c**) and iron agglomerates (**d**).

Aluminosilicate layers are known to roll in cylinders as a result of three main effects: (i) the strain caused by lattice mismatch between adjacent silicone dioxide and aluminum oxide sheets [41]; (ii) the attraction between the interlayer hydroxyl groups in octahedrons [42]; and (iii) the surface tension of water [43]. These structures are also observed in Dunino clay (Figure 2d,e), with walls thickness ranging from 10 to 50 nm. Rolled halloysite looks more unstable under an electron beam than the platy one, making image acquisition difficult. During high-resolution acquisition, the ordered gap in the c direction tends to gradually disappear after a few seconds. Using a decreased electron dose, it was possible to document the presence of some large and facet rolled structures with thick and compact walls (Figure 2d), but also some thin and delicate structures, composed of a few atomic stacked layers (Figure 2e). All the measurements of the c-axis gap in the last class of structures provided values very close to 10 Å (Figure 2e,f). Randomly distributed agglomerates of spherical particles were observed in different areas of the sample (Figure 2g). Higher magnification images of these particles showed that they were crystalline, porous and with dimensions below 20 nm (Figure 2h). The electron diffraction pattern revealed that these nanoparticles were formed by iron oxide with a hematite α-Fe_2_O_3_ structure (Figure 2i). The hematite structure is a hexagonal crystal system consisting of iron atoms surrounded by six oxygen atoms. The hematite exhibits C3v symmetry [44] within two different FeO bond lengths. In α-Fe_2_O_3_, the oxide ions (O_2_^−^) are arranged along the (0 0 1) plane of a hexagonal closed-packed lattice, whereas two-thirds of the octahedral interstices are occupied by the cations (Fe^3+^) in the (0 0 1) basal planes. The tetrahedral sites are unoccupied. This cationic arrangement generates pairs of FeO_6_ octahedrons, in which the edges are shared by three neighboring octahedrons in the same plane and one face with an octahedron in an adjacent plane in the (0 0 1) direction [44]. Iron could be present, not only as fine oxide particles, but also as Fe^3+^ substitutional to Al^3+^ in the layers [45] as also confirmed by chemical characterization determined by EELS spectroscopy. The acquired spectra are reported in Figure 3.

The EELS spectra shown above report the K-edge of oxygen, aluminu m, and silicon at 532 eV, 1560 eV, and 1839 eV, respectively, and the L-edge of iron at 723 eV. These peaks confirmed the presence of silicon, aluminum, and iron in the platy structures (Figure 3a) and confirmed that agglomerations of nanoparticles were formed by iron oxide (Figure 3b), as also observed in the chemical maps reported in Figure S3. In particular, Figure S4 of the Supplementary Information reports the O and Fe chemical maps on a small area of a platy structure, and the corresponding EELS spectra acquired from three different points of the same area. The brighter signal was associated with small spherical particles of iron oxide, as confirmed by EELS spectrum (red curve); iron in the lower content was still present at other points of the same layer, as shown by the blue and green spectra, respectively.

To sum up, iron was largely present in the Dunino sample and was distributed all over the sample; its content influenced the particle morphology [46]: a higher amount of iron, mainly as oxide nanoparticles, was observed on the surface of the platy structures. Furthermore, the b parameter measured by electron diffraction of these structures was larger than the one reported in the literature [47], confirming the presence of substitutional iron in the layers. In the rolled structures, the iron amount was low enough (<1% as reported by EDX analysis shown above) to allow the layers to roll up.

### 3.2. Measurement of Point of Zero Charge (PZC)

**Figure 4:**
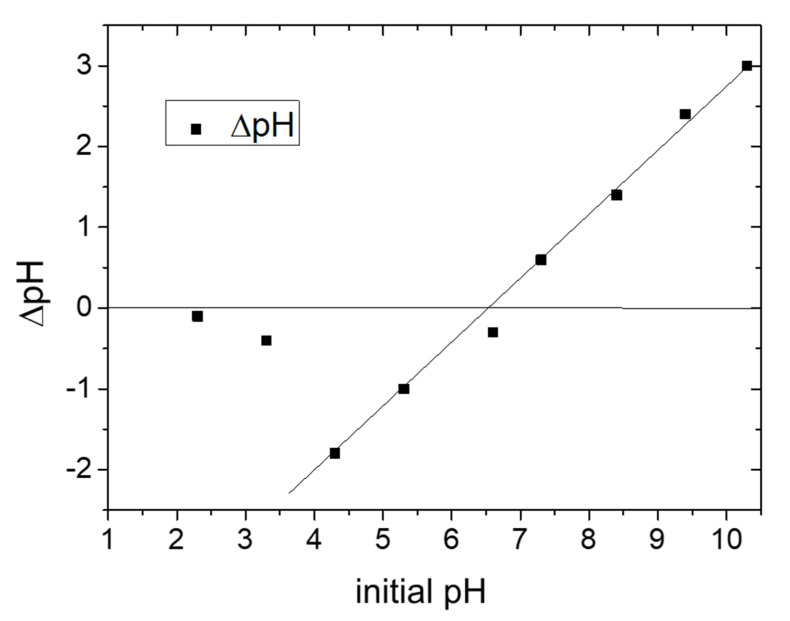
pH variation vs. initial pH value for the determination of PZC of the clay.

The point of zero charge (pH_PZC_) of the raw halloysite mineral was determined by the procedure reported in Section 2. Figure 4 reports the pH change (∆pH = pH_initial_ − pH_final_) of the solutions after the addition and equilibration of clay vs. the initial pH.

For an initial pH lower than 6.5, ∆pH is negative (pH_fin_ > pH_in_) due to the capture of protons by the halloysite surface (protonation of halloysite); in this pH range, a larger change was observed for pH = 4.3.

For initial pH higher than 6.5, ∆pH is positive (pH_fin_ < pH_in_) due to proton release from the clay surface. At pH = 6.5, no variation was observed after adding halloysite to the solution and this was the point of zero charge.

### 3.3. Adsorption Experiments

Considering its structure, halloysite could adsorb ions and neutral molecules by (i) ion exchange at the negatively charged sites in the tetrahedral sheets, or (ii) by the formation of surface complexes with both Al–OH and Si–OH groups located at the layer edges. Therefore, we tested the adsorption properties of Dunino raw halloysite mineral for positive and negative dyes, i.e., MB and MO, respectively (the structures of MO and MB are reported in Figure S5). The adsorption selectivity of this clay was also investigated by mixing MB- and MO-dye solutions. Figure S6 of the Supplementary Information reports the UV-Visible reference spectra of the clay dispersion without any dye molecules (a), of dye solutions without clay (b), and of kaolin/dye dispersion after few minutes of contact (c). The UV-Visible spectra of the clay at different concentrations are the sum of the extinction contributions (absorbance and scattering) due to both aluminosilicate layers and iron oxide NPs.

MB is a cationic, thiazine dye, which absorbs light in a band centered at 664 nm (*n*-ð *) (monomer) with a shoulder at 610 nm, corresponding to the MB dimer (see Figure S6b of the Supplementary Information). Higher MB aggregates occurred at high concentrated solutions and are easily detectable by the appearance of an absorption band at lower wavelengths with respect to the monomer [12]. The UV-Vis absorbance spectrum of MO dissolved in water shows two maxima: the first at 270 nm and the second at 465 nm, related to the benzene ring in MO and the azo linkage of MO [9], respectively (see Figure S6b of the Supplementary Information). The latter absorbance peak was used to quantify the MO concentration reduction or degradation due to adsorption and photocatalysis; any variation of the 270 nm peak position was also correlated with the formation of by-products because of the azo-dye degradation. In mixed solutions, the UV-Visible spectrum is the sum of the spectra of the two components: the two maxima at 664 nm (for MB) and 465 nm (for MO) are still present and the peak at 290 nm is the sum of both contributions (see Figure S6b of the Supplementary Information). The mixed solution is stable after three hours (see Figure S6b).

#### 3.3.1. Removal of MO and MB

**Figure 5:**
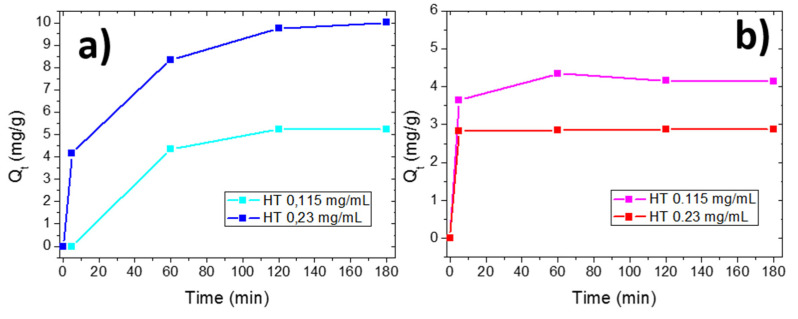
Q_t_ values for MB (**a**) and MO (**b**) adsorption at different clay concentrations.

The UV-Vis absorbance spectra of halloysite dispersions with two concentration values (0.115 mg/mL and 0.23 mg/mL) in the presence of dyes (10^−5^ M) were recorded versus contact time. The UV-Visible reference spectra of halloysite/dye solutions after a few minutes of contact are reported in the Supplementary Information (see Figure S6c). No variations in the shapes of the dye absorbance peaks were observed after addition of the clay powder and the removal efficiency was calculated by the reduction of the absorbance peaks characteristic of MO and MB, respectively, from their maximum intensity values. The amounts of adsorbed mg of dyes per gram of clay (Q_t_) versus time of contact for the two different clay concentrations are reported in Figure 5. Dyes were quickly adsorbed after few minutes of being in contact with the clay powder; after this time, MB adsorption increased with an increase in both contact time and clay concentration, while MO was not further adsorbed on clay, independently from the clay concentration (see Figure 5a,b, respectively). The positive MB charge favors its adsorption on clay, occurring via electrostatic attraction and this adsorption increased with contact time and clay concentration. At the end of the experiment (*t* = 180 min), the lowest concentrated clay dispersion adsorbed the 19% of the initial MB molecules (corresponding to a Q_t_ of 5.3 mg/g); by doubling the clay concentration, 70% of the MB molecules were removed (corresponding to Q_t_ of 10 mg/g).

**Figure 6:**
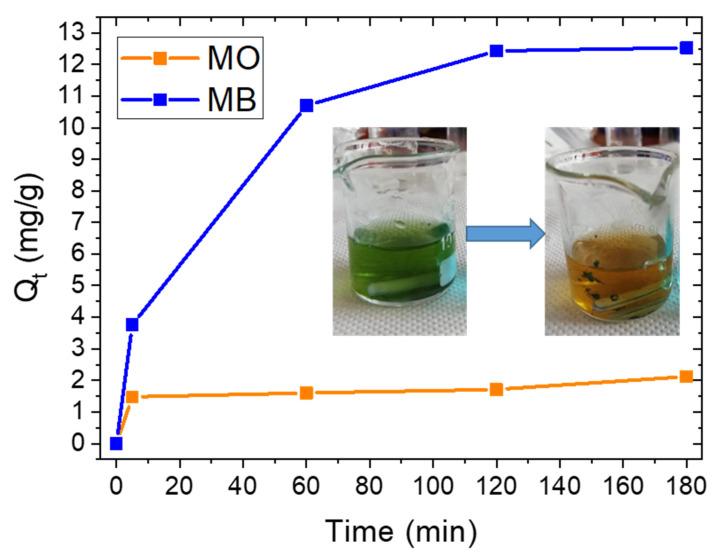
Q_t_ values for MB and MO adsorption in the green solution for a 0.23 mg/mL clay concentration. The photo of the green solution before and after the process is reported in the inset, clearly showing the change of the color due to the selective MB removal.

In the case of MO molecules, the maximum adsorption occurred in the first few minutes. The negative charge does not favor their adsorption on clay due to electrostatic repulsion, but other different interactions, such as the formation of surface complexes with both Al–OH and Si–OH groups located at the layer edges, H-bonding interactions and *n*–π interactions took place. For this reason, after a few minutes, the few available adsorption sites were occupied and no further adsorption occurred. After 180 min, the MO Q_t_ value was 4.13 mg/g, corresponding to a removal of 10% of the initial dye concentration for the lowest halloysite concentration; for the highest halloysite concentration, the MO Q_t_ value was 2.88 mg/g, still corresponding to a dye removal of 10%. The lower MO Q_t_ value observed for 0.23 mg/mL halloysite concentration with respect to the one observed for 0.115 mg/mL can be addressed by the clay precipitation phenomenon taking place in the MO solution due to electrostatic repulsive forces. The adsorption selectivity of raw halloysite mineral was investigated by adsorption of MO and MB molecules in mixed solution (1:1), that is the Green solution. Figure 6 reports the Q_t_ values for MO and MB in mixed solutions, in contact with halloysite at a higher concentration (0.23 mg/mL). It is possible to observe (in the inset) the change in color from green to orange, due to the selective MB removal.

The spectrum of Green is a combination of the MO and MB spectra and is reported in the Supplementary Information (Figure S6b). After the dispersion of halloysite powder in the Green solution, the MO concentration decreased slightly at the beginning and then did not change further with contact time. On the contrary, MB molecules were adsorbed mainly in the first hour, with a slower adsorption occurring between the first and the second hours. No further adsorption was observed from the second to the third hour, as confirmed by Q_t_ values reported in Figure 6. Furthermore, in the Green solution, we observed the formation of flocculates in the first hour, probably due to fast adsorption phenomena not observed in the case of MB solution.

Table 2 reports the percent of removal for each dye at different times, obtained by evaluating the decrease of the peak intensity at 469 nm and at 664 nm, respectively for MO and MB, in mixed solution. Additionally, in this case, the amount of removed MO molecules is lower than the amount of removed MB molecules: in particular, after three hours, 89% and 19% of MB and MO were removed, respectively, corresponding to Q_t_ values of 12.53 mg/g for MB and 2.14 mg/g for MO. These results point to the selectivity of this clay towards positively charged molecules.

An interesting finding is that the removal efficiency of MB increased in mixed solution with respect to pure MB solution, after three hours. This is shown in Table 3, where a comparison of the removal efficiency values, expressed as MO and MB removal (%) and Q_t_, by the same amount of clay are reported for single-dye solutions and for mixed solutions.

In the mixed solution, this clay confirmed a preferential adsorption for positively charged molecules with respect to negative ones within a higher removal efficiency for this dye with respect to a single-dye solution. This effect can be explained by performing z-potential measurements and measuring the surface charge.

Our adsorption experiments were conducted at pH 5.8 and, according to the graph reported in Figure 4, for this pH value (lower than 6.5), halloysite is negatively charged. An electrostatic interaction can explain the much larger adsorption of MB with respect to MO. This was also confirmed by z-potential measurements, i.e., a z-potential value of –50.87 mV was measured for this clay dispersed in water at pH 5.8. This value was also measured in the presence of dyes: (i) when MO was added to the clay dispersion, it was not possible to have a reliable measurement since clay nanoparticles aggregated due to the negative charge of MO. This aggregation reduced the surface area available for adsorption, resulting in a lower Q_t_ value by increasing the clay concentration (see Figure 5b); (ii) in presence of MB, MB adsorption occurred (see Figure 5a) and clay z-potential increased to 35.1 mV, underlining that dye molecules were adsorbed on the clay surface; and (iii) the same effect was observed for mixed dye solutions, where the z-potential increased to 28.83 mV. Furthermore, since this value was less positive than the one measured in MB solution alone, we can deduce that this is a consequence of the combined interactions with MB and MO.

#### 3.3.2. Adsorption Kinetics

**Figure 7:**
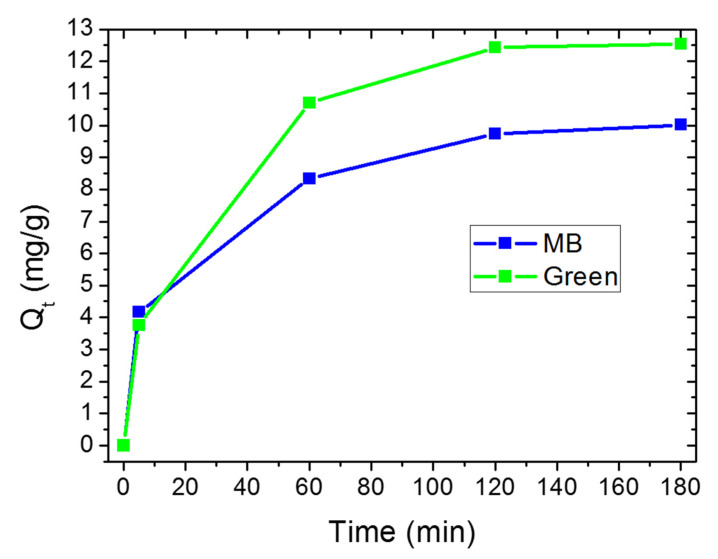
Amount of adsorbed MB molecules in mg per gram of raw halloysite as a function of time, in MB and Green solutions.

In order to investigate the adsorption mechanism, kinetic constants were determined; furthermore, kinetic analysis is useful from a practical point of view to determine the time required to complete the adsorption process. We do not consider MO since its adsorption is low and it occurred mainly in the first hour. For what concerns MB, we report a comparison on the kinetic mechanisms for MB adsorption in MB or mixed solution by the highest clay concentration. As for MO, at the lowest clay concentration, the adsorption of MB is too low and fast for an accurate evaluation of kinetic mechanisms. Figure 7 reports the amount of adsorbed MB molecules, Q_t_ (*t* mg per gram of clay), as a function of time, for the 0.23 mg/mL clay concentration.

For both the solutions, MB adsorption occurred suddenly in a few minutes and then it slowed down, as evidenced by the slope of the curves in Figure 7. The adsorption in the first few minutes was quite similar for both solutions, in terms of both Q_t_ and kinetic constants. In the first hour, the clay continued to adsorb MB molecules, with a higher amount and velocity in the case of mixed solution with respect to the MB solution. In the second and third hours, the adsorption still occurred for both solutions, reaching an equilibrium at the maximum Q_t_, i.e., 10 and 12.53 for MB and Green solution, respectively.

Different kinetic models can be used for studying the adsorption phenomena. Some models, such as the pseudo-first-order (PFO) or pseudo-second-order (PSO) models, rely on the fact that the sorption is the rate-limiting step (adsorption reaction models) and these are usually suitable to explain chemisorption [40,41,42,43,44,45,46,47,48]. According to these models, adsorption depends on the availability of adsorption sites on the surface of the adsorbent, rather than on the adsorbate concentration in the bulk solution [48].

The linear expression of PFO is reported in Equation (1).

(1) $$\ln\left(Q_{e}\;–{\;Q}_{t}\right)={\mathrm{lnQ}}_{e}\;–{\;k}_{1}t$$

where Q_t_ is the amount of adsorbate adsorbed onto the adsorbent at time *t* (mg/g), Q_e_ is the equilibrium adsorption capacity (mg/g), and k_1_ is the rate constant (min^−1^). The value of k_1_ is determined by plotting ln(Q_e_−Q_t_) versus *t*.

The linear expression of PSO is reported in Equation (2).

(2) $$\frac{t}{Q_{t}}=\frac{1}{k_{2}Q_{e}{}^{2}}+\frac{t}{Q_{e}}$$

where Q_t_ is the amount of adsorbate adsorbed onto the adsorbent at time *t* (mg/g), Q_e_ is the equilibrium adsorption capacity (mg/g), and k_2_ is the rate constant per min. The value of k_2_ is determined by plotting *t*/Q_t_ versus *t*. This model has been widely applied in order to describe chemisorption involving covalent forces and ion exchange between the adsorbent and adsorbate [40]. With respect to the PFO model, the PSO one has the advantage of directly calculating equilibrium capacity Q_e_ [48].

The adsorption of solute from a solution into a sorbent involves mass transfer of adsorbate (film diffusion), surface diffusion, and pore diffusion. In this regard, other kinetic models like intra-particle (IP) model consider the diffusion as the rate-limiting step (adsorption diffusion models) and these are well suited with physisorption processes [49]. The intraparticle diffusion equation, as described by Weber and Morris [50], is reported as Equation (3).

(3) $$Q_{t}=k_{d}\left(t^{0.5}\right)+\;C$$

where Q_t_ is the amount of adsorbate adsorbed onto the adsorbent at time *t* (mg/g), k_d_ is the rate constant (mg/g)·min^0.5^, and C determines the boundary layer effect (i.e., higher values, larger film diffusion resistance) and is linked to external mass transfer [49,50].

Independently from the kinetic model, the linear forms are applied in order to study adsorption kinetics, and the suitability of any model depends on the degree of linear correlation between the experimental and the predicted values (R^2^) [13]. Figure S7 of the Supplementary Information reports the linear expression of each kinetic models according to Equations (1)–(3), in which the Q_t_ values versus time were obtained by considering the absorption peak at 664 nm for experiment conducted in MB and Green solution using clay power at a concentration of 0.23 mg/mL. Table S2 of the Supplementary Information reports R^2^ values obtained by fitting the experimental data of Figure S7 for MB molecules removal by clay in both MB and Green solutions, according to the pseudo first order kinetic model, the pseudo-second-order kinetic model, and the intraparticle diffusion model, respectively. For all the investigated solutions, higher R^2^ values were obtained by fitting the data with the pseudo second order kinetic model, confirming that the MB adsorption occurred as chemisorption involving covalent forces and ion exchange between the adsorbent and adsorbate. The related fitting parameters for this model and the two solutions are reported in Table 4.

In both cases, besides the highest R*^2^* values, the calculated adsorption capacities (Q_e_) are close to experimental data (Q_t_ of Table 4), indicating that this model is more suitable to describe the adsorption of MB on the studied samples. The adsorption kinetic constant of MB molecules in Green solution is the double with respect to MB solution itself: the presence of MO increased the adsorption abilities of clay towards positive charged molecules in terms of either selectivity, removal efficiency and kinetic constant.

#### 3.3.3. Adsorption Isotherms

Adsorption isotherms are of great importance in the design of adsorption systems since they provide information, such as the capacity of the adsorbent or the amount required for removing a unit mass of pollutant under the investigated conditions. For this purpose, MB adsorption experiments in single dye or mixed dyes solutions were conducted on the halloysite mineral by varying the initial MB concentration (3.2, 6.4, 9.6, 12.8 mg/L), as described in Section 2. UV-Vis spectra were acquired after the equilibrium was reached.

Equilibrium data are usually investigated using two models [51]. The Freundlich isotherm is the earliest known relationship describing a multi-layer process in which the amount of adsorbed solute per unit adsorbent mass increases gradually. The Freundlich isotherm may be written as follows:

(4) Qe=(Ce)1n+ KF

and its linear expression is:

(5) $${\mathrm{logQ}}_{e}=\frac{1}{n}\log C_{e}+{\;\mathrm{logK}}_{F}$$

where K_F_ is the constant of the Freundlich isotherm (L^1/*n*^ mg^(1−1/*n*)^/g), and 1/*n* is the Freundlich exponent. This linear form of the equation can be used to evaluate whether the adsorption process satisfies the Freundlich isotherm and to identify the constants. Higher value for K_F_ indicates higher affinity for adsorbate and the empirical parameter 1/*n* values lying between 0.1 < 1/*n* < 1 indicate favorable adsorption. K_F_ and 1/*n* can be determined from the linear plot of logQ_e_ versus logC_e_, respectively.

Langmuir-type adsorption is considered to describe a monolayer process: once a dye molecule occupies a site, no further adsorption can take place at that site and there is no interaction between the adsorbate molecules.

The linear expression of the Langmuir model is given as:

(6) $$\frac{1}{Q_{e}}=\frac{1}{K_{L}+Q_{\max}}\cdot \frac{1}{C_{e}}+\frac{1}{Q_{\max}}$$

where *C_e_* is the solute (adsorbate) concentration in the solution at equilibrium (mg/L), *Q_e_* the solute mass adsorbed per unit adsorbent mass at equilibrium (mg/g), *K_L_* the constant of the Langmuir isotherm (L/mg), and *Q_max_* relates to the maximum adsorption capacity (mg/g). In any adsorption experiments, *C_e_* can be measured and *Q_e_* can be calculated for a series of different conditions. Then *Q_max_* and *K_L_* constants are calculated from the slope and intercept of the plot of Equation (6). The maximum adsorption capacity per unit adsorbent mass (*Q_max_*) is determined along with the Langmuir constant K_L_ showing the solute affinity to the adsorbent.

Table S3 in the Supplementary Information reports the amount of adsorbed dye on unit mass of clay at equilibrium for increasing MB concentrations, in both single dye or mixed dyes solutions. Analogously to the adsorption kinetics results, we do not show the MO adsorption isotherms since the agreement with the Langmuir and Freundlich models is not satisfactory (R^2^ << 0.9) due to low affinity with the clay surface.

The amount of adsorbed dye molecules increased with initial MB concentration (C_0_) and the higher value was always observed for MB in mixed dyes solutions, for each investigated concentration.

Both the Freundlich and Langmuir isotherm models for MB adsorption in single or mixed solution at a temperature of 298K are illustrated in Figure S8. Parameters and correlation coefficients obtained from these models are summarized in Table 5.

According to the values of 1/*n* reported in previous table, the adsorption of MB is favored both in single-dye and mixed-dye solutions. However, it was found that the fitting to the Langmuir model gave higher values of correlation coefficients (R^2^) than those for the Freundlich model at the temperature investigated. In other words, the Langmuir model is better than Freundlich model in describing the behavior of MB adsorption onto halloysite clay for both MB alone and in mixed solutions.

The Q_max_ values for MB adsorption on Dunino clay is 47.46 mg/g and it doubles in the presence of MO, confirming the kinetic analysis results. The K_L_ values for MB adsorption is slightly higher in the presence of MO with respect to MB itself. In addition, a dimensionless constant separation factor or equilibrium parameter R_L_ is also reported.

R_L_ is defined by the following equation:

(7) $$R_{L}=\frac{1}{1+K_{L}C_{0}}$$

where *K_L_* (L/mg) is the Langmuir constant and *C*_0_ (mg/L) is the initial MB concentration. This parameter allows to determine if the adsorption process is favorable or unfavorable. The *R_L_* value indicates the adsorption process is irreversible when it is 0; favorable when *R_L_* is between 0 and 1; linear when *R_L_* is 1; and unfavorable when *R_L_* is greater than 1.

Table S4 in the Supplementary Information reports the R_L_ values for MB adsorption in single dye or mixed dyes solutions by increasing MB concentration. The R_L_ values ranged between 0.6646 and 0.3313 for MB itself and between 0.6788 and 0.3457 for MB adsorption in presence of MO. These values confirm that the adsorption process is favorable.

#### 3.3.4. Adsorption Mechanism

The experimental data obtained are in good agreement with current knowledge on the adsorption properties of kaolin minerals [11,19,20]. We measured the total surface charge of dispersed raw halloysite mineral by z-potential measurements and the evaluation of pH_PZC_, as reported in Section 3.2 and we found that its PZC was at pH 6.5, thus the surface was negative at a pH lower than pH_PZC_. Thus, in our experiments conducted at pH = 5.8, cationic species were attracted by the sorbent surface via electrostatic forces and were adsorbed in higher quantities than anionic ones. This explains the adsorption selectivity of this clay toward MB with respect to MO. Indeed, by increasing contact time and halloysite concentration, MB adsorption on the clay surface increased because of a higher amount of negative charged adsorption sites. On the contrary, the limited MO adsorption shown by halloysite in the first minutes could be ascribed to different interactions such as the formation of surface complexes with both Al–OH and Si–OH groups located at the layer edges, H-bonding interactions and *n*–π interaction. These interactions are less intense and probable to occur than electrostatic ones, explaining the reduced adsorption of MO with respect to MB (see Figure 5b).

According to kinetic analysis, the PSO kinetic model best fits the experimental data for MB adsorption in both MB and mixed solutions underlining that the studied adsorption process occurred mainly as chemisorption, involving covalent forces and electrostatic interactions between the adsorbent and adsorbate. This evidence is in agreement with our explanation of MB adsorption on the negative surface of halloysite. We have found that the MB adsorption process can be described by Langmuir isotherm model, confirming the high affinity of this clay for the cationic dye, as evidenced by K_L_ and R_L_ values. Furthermore, we showed that in mixed solution, the presence of MO favors the adsorption of MB molecules, in particular considering the kinetic constant and the Q_max_ value. This could be ascribed to different reasons such as dispersion of clay layers in solution and the acid character of methyl orange that favors the deprotonation of silanol and aluminol, resulting in higher negative charge and thus higher MB adsorption. MO added to MB/clay solution increases the ionic strength of the solution generating three main effects:

i. a decreasing of surface potentials according to [52] and, thus, an increase in the MB concentration gradient on the clay surface, resulting in a higher MB adsorption;
ii. as an effect of the acid character of MO, the alumina faces and edges, deprotonation reactions occurred, with an increase in the clay negative surface charge, resulting in an increase in the MB adsorption sites on the clay surface;
iii. the increasing of ionic strength also results in an increasing of clay cluster dimension [52], as evidenced by the appearance of flocculates in mixed dyes/clay solution not observed in MO/clay or MB/clay solution respectively.

Finally, we report in Table 6 a comparison of our results with the ones reported in the literature for halloysite (HT) or Kaolinite clay used for MB removal.

Considering both the Q_t_ values at equilibrium and the one obtained by isotherm analysis, our results are better or comparable to the ones reported in the literature, with the advantage that our material has not been treated for purification or activation unlike the materials tested in the literature and reported in Table 6.

#### 3.3.5. Regeneration of Halloysite

**Figure 8:**
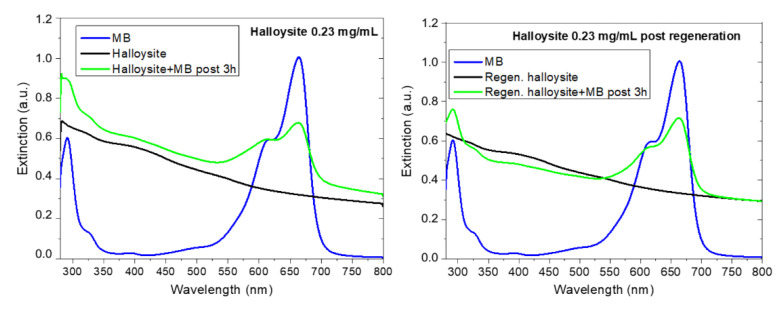
UV-Visible extinction spectra of raw halloysite (**a**) and regenerated halloysite (**b**) dispersions before and after being in contact with MB for three hours. In both graphs the MB reference spectra are reported (blue spectra) for comparison.

After a 3h MB adsorption process, halloysite was regenerated, as described in Section 2. The UV-Vis spectra of clay dispersions and MB/clay dispersions after three hours before and after regeneration were acquired and are reported in Figure 8a,b, respectively. Both the adsorption processes (i.e., before and after regeneration) were conducted using the same halloysite and MB concentrations.

Figure 8a refers to the first adsorption experiment reporting the UV-Vis spectra before (black curve) and after (green curve) MB addition to the clay dispersion. The clay adsorbed MB and a Q_t_ value of 10 mg/g, was measured, as reported in Table 6. The powder was recovered and regenerated by heating until its color turned from blue to the original one. The regenerated powder was dispersed in water and its UV-Vis spectrum is reported in Figure 8b (see black curve). The MB peak at 664 nm is not visible anymore, confirming the removal of adsorbed dye from the clay after heating. The regenerated material was tested again for the removal of MB: the spectrum acquired after three hours (see green curve of Figure 8b) confirms the ability of regenerated clay to adsorb again MB (as evidenced by the peak at 664 nm). The adsorption efficiency is slightly reduced (Q_t_ = 8.5 mg/g) after the regeneration process, but the regeneration process can be optimized further.

## 4. Conclusions

In this paper, the use of natural halloysite from Dunino is reported for the selective and efficient removal of positive charged dye. As confirmed by morphological and chemical characterization, the natural clay consists in platy structures and tubular ones mainly composed by Si, Al, O, and Fe. Iron is distributed all over the sample both as substitutional, and as hematite in a total amount of 14% as measured by XRF analysis. In particular, iron, mainly as oxide nanoparticles, was observed on the surface of platy structures. In rolled or few layers structures, the iron is low, but still present, as evidenced by the higher b parameter measured on these structures with respect to the one reported in literature [40].

This clay has a negative surface charge at neutral pH, as confirmed by z-potential measurements and the evaluation of pH_PZC_, and this surface charge is the main responsible of its adsorption properties. Indeed, the adsorption of anionic dye i.e., MO on this clay is low at neutral pH, while good results were obtained in removal of cationic dye, i.e., MB from both MB solution and mixed MO/MB solution. The observed values of MB molecules adsorbed for gram of clay are better or comparable with previous results reported in the literature for the adsorption of MB on raw or purified/activated kaolin (see Table 6). According to kinetic analysis, the pseudo second order kinetic model best fits the experimental data for MB adsorption in both MB and mixed solutions underlining that the studied adsorption process occurred mainly as chemisorption, involving covalent forces and ion exchange between the adsorbent and adsorbate. The isotherm adsorption plots showed that the adsorption process can be fitted very well by the Langmuir model. According to this model, the process is a monolayer process involving a direct interaction of single dye molecule within sites on clay surface: when a molecule occupies a site, no further adsorption can take place at that site and there is no interaction between the adsorbate molecules. The K_L_ and R_L_ values confirmed a high affinity of MB for this clay favored by the presence of MO.

The raw halloysite mineral has shown to be selective toward the adsorption of MB with respect to MO and the adsorption rate is enhanced in mixed dyes solution. This effect could be explained considering the acid character of MO and the raising of the solution ionic strength when MO is added to MB/clay solution. As a consequence, an increase of the surface charge and potential of halloysite clay is achieved, producing a higher MB gradient concentration from the solution to clay surface with an enhancement of the MB adsorption.

The possibility to regenerate halloysite after dye adsorption and reuse it for new adsorption processes with similar performance was also shown.

In conclusion, our results confirm the possibility of using Dunino clay as cheap, efficient, reusable and selective sorbent for positively charged molecule removal from water. The surface properties of this clay being dependent on the pH and ionic strength of the solution point out the possibility of engineering specific adsorbents for the removal of water contaminants from natural resources, saline water or seawater.

## Figures and Tables

**Table 1:** Chemical composition of halloysite measured by XRF.

Element	Mass (%)
Si	18.76
Al	16.62
O	45.15
Fe	14.20
Ti	1.96
Ca	0.74
K	0.68
Na	0.54
P, Mn, Mg, Cr	0.1 < x< 0.5
Ni, Zn, As, Zr, Co, V, S, Cu	0.01 < x < 0.1
Y, Sr, Rb	<0.01

**Table 2:** MB and MO removal (%) in mixed-dye solution by raw halloysite mineral (concentration: 0.23 mg/mL) at different contact times.

Dye-Peak (nm)	Removed Dye (%)			
	5 min	1 h	2 h	3 h
MO-469	16	15	15	19
MB-664	25	76	88	89

**Table 3:** MB and MO removal (%) and mg of molecules adsorbed by gram of clay (Q_t_) in single MO, MB and Green solution, respectively, after three hours, for a clay concentration of 0.23 mg/mL.

Adsorption Efficiency	MB	MB (Green)	MO	MO (Green)
Q_t_ (mg/g)	10.0	12.5	2.9	2.1
Removal (%)	70	80	10	15

**Table 4:** Intercept, slope, Q_e_ values and kinetic constants calculated according to the pseudo second order kinetic model for the removal of MB molecules by clay in MB or Green solution, respectively.

Dye Solution	Pseudo Second Order t/Q_t_ = (1/Q_e_)t + (1/kQ_e_^2^)			
	Intercept	Slope	Q_e_ (mg/g)	k (g mg^−1^ min^−1^)
MB	1.03 ± 0.35	0.095 ± 0.003	10.55	0.009
Green	0.42 ± 0.45	0.085 ± 0.004	12.77	0.017

**Table 5:** Langmuir and Freundlich plotting parameters for MB adsorption on clay surface in single and mixed solution.

Solution	Langmuir Isotherm			Freundlich Isotherm		
	Q_max_ (mg/g)	K_L_ (L/mg)	R^2^	1/*n*	K_F_ (L^1/n^mg^1−1/*n*^/mg)	R^2^
MB	47.46	0.148	0.997	0.676	1.050	0.979
MB_Green	89.13	0.158	0.986	0.717	12.512	0.924

**Table 6:** Comparison of MB adsorption abilities for clays used in this work and for similar materials reported in the literature. (n.a. = not available, data not reported).

Clay Sample	Dye Concentration (mg/mL)	Q_t_ (mg/g)	Q_max_ (mg/g) (Langmuir Isotherm)	Ref.
Raw halloysite (Dunino cave)	[MB] = 3.2–12.8	10.0 (@3.2 mg/mL, *t* = 3 h)	47.46	This work
Raw halloysite (Dunino cave)	[MB]_Green_ = 3.2–12.8	12.5 (@3.2 mg/mL, *t* = 3 h)	89.13	This work
Tubular halloysite (Morocco) acid treated in HCl	[MB]= 5 [MB] = 20	4.5 16.0	n.a.	[26]
Raw kaolinite (Morocco)	[MB] = 10	7.5		
Halloysite clay (Henan Province, China) treated by HCl	[MB] = 50–500	n.a.	91.32 (298 K) 96.34 (308 K) 103.63 (318 K)	[27]
Raw kaolin (Silonijan, India)	[MB] = 12–25	n.a.	13.99	[28]
Kaolin (Silonijan, India) treated with H_2_O_2_	[MB] = 12–25	n.a.	15.55	
Raw kaolin (Silonijan, India) after calcination	[MB] = 12–25	n.a.	7.59	
Kaolin (Silonijan, India) treated with H_2_O_2_ and calcinated	[MB] = 12–25 mg/L	n.a.	8.88	
Raw kaolin (Silonijan, India) treated with NaOH	[MB] = 12–25 mg/L	n.a.	16.34	
Kaolin (Silonijan, India) treated with H_2_O_2_ and NaOH	[MB] = 12–25 mg/L	n.a.	20.49	
Kaolinite (Ajax Chemicals, Sydney) washed in acid		n.a.	33.00	[29]
Commercial kaolin KOM manufactured by Surmin-Kaolin (Nowogrodziec, Poland)	[MB] = 6–20 mg/L	n.a.	12.90	[30]

## Data Availability

The data presented in this study are available on request from the corresponding author.

## Supplementary Materials

The following are available online at https://www.mdpi.com/article/10.3390/ma14133676/s1, Figure S1: SEM image of raw halloysite powder deposited on a polymeric substrate for EDX analysis. The red and green squares indicate the sample areas (tube and plate, respectively) where EDX spectra were acquired. Table S1. wt.% of elements acquired by EDX spectra on two different area of the deposit. Figure S2: Scheme of clay crystal structures. Figure S3: Dark field STEM images and chemical maps acquired on raw halloysite powder. Figure S4: Chemical maps acquired on a platy structure and corresponding EELS spectra acquired on three different points. Figure S5: Chemical structures of Methyl Orange (on the left) and Methylene Blue (on the right) molecules. Figure S6: (a) UV-Visible spectra of raw halloysite powder dispersed in water at two different concentrations. (b) UV-Visible spectra of MO, MB and Green solutions (i.e., orange, blue and green curves, respectively). The spectrum for Green solution was acquired also after three hours. (c) UV-Visible spectra of MO, MB and Green solutions after few minutes of contact with halloysite powder at different concentrations. Figure S7. Linear expression of pseudo first order kinetic model (a), pseudo second order kinetic model (b) and intraparticle diffusion model (c) for MB adsorption in both MB and Green solutions (blue and green curves, respectively). It is clear, by a simple visual inspection, that only in case (b) a reliable linear correlation of the experimental data is evidenced. Table S2: R^2^ values obtained by fitting the experimental data for MB removal for raw halloysite concentration of 0.23 mg/mL for MB and Green solutions, using the pseudo first order kinetic model, the pseudo-second-order kinetic model and the intraparticle diffusion model. Table S3: Amount of adsorbed dye on unit mass of clay at the equilibrium for increasing MB concentration both in single dye or mixed dyes solution. Figure S8: Langmuir (a,c) and Freundlich (b,d) plots for MB adsorption on Dunino clay in single (a,b) and mixed solution (c,d). Table S4: R_L_ values obtained by Langmuir plots for MB adsorption in single and mixed dyes solution changing the MB initial concentrations.
