# Supplementary material for: Structural Characterization and Adsorption Properties of Dunino Raw Halloysite Mineral for Dye Removal from Water
Source: Materials (Basel). 2021 Jul 1;14(13):3676. doi: 10.3390/ma14133676 (PMC8269871; doi:10.3390/ma14133676)
Supplement: Supplementary file 1 [file materials-14-03676-s001.zip › materials-1198283-Supplementary.pdf]

materials

Supplementary

Structural Characterization and Adsorption Properties of Dunino Raw Halloysite Mineral for Dyes Removal from Water

Simona Filice [1], Corrado Bongiorno [1], Sebania Libertino [1], Giuseppe Compagnini [2], Leon Gradon [3], Daniela Iannazzo [4], Antonino La Magna [1] and Silvia Scalese [1,*]

[1] Istituto per la Microelettronica e Microsistemi, Consiglio Nazionale delle Ricerche (CNR-IMM), Ottava Strada n.5, I-95121 Catania, Italy; simona.filice@imm.cnr.it (S.F.); corrado.bongiorno@imm.cnr.it (C.B.); sebania.libertino@imm.cnr.it (S.L.); antonino.lamagna@imm.cnr.it (A.L.M.)
[2] Dipartimento di Scienze Chimiche, Università di Catania, Viale A. Doria 6, 95125 Catania, Italy; gcompagnini@unict.it
[3] Faculty of Chemical and Process Engineering, Warsaw University of Technology, ul. Warynskiego 1, 00-645 Warsaw, Poland; Leon.Gradon@pw.edu.pl
[4] Dipartimento di Ingegneria, Università degli Studi di Messina, Contrada di Dio, I-98166 Messina, Italy; diannazzo@unime.it
* Correspondence: silvia.scalese@imm.cnr.it

Citation: Filice, S.; Bongiorno, C.; Libertino, S.; Compagnini, G.; Gradon, L.; Iannazzo, D.; La Magna, A.; Scalese, S. Structural Characterization and Adsorption Properties of Dunino Raw Halloysite Mineral for Dye Removal from Water. *Materials* **2021**, *14*, 3676. https://doi.org/10.3390/ma14133676

Academic Editors: Alain Celzard/Bo Xiao

Received: 10 April 2021
Accepted: 28 June 2021
Published: 1 July 2021

Publisher's Note: MDPI stays neutral with regard to jurisdictional claims in published maps and institutional affiliations.

Copyright: © 2021 by the authors. Licensee MDPI, Basel, Switzerland. This article is an open access article distributed under the terms and conditions of the Creative Commons Attribution (CC BY) license (http://creativecommons.org/licenses/by/4.0/).

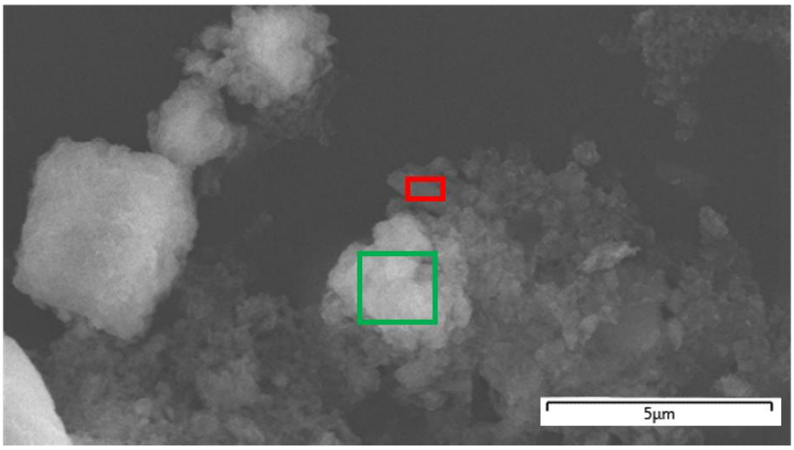

Figure S1. SEM image of raw halloysite powder deposited on a polymeric substrate for EDX analysis. The red and green squares indicate the sample areas (tube and plate, respectively) where EDX spectra were acquired.

Table S1. wt.% of elements acquired by EDX spectra on two different area of the deposit.

Element	wt. % (Red Area)	wt. % (Green Area)
**C**	51.2	24.0
**O**	34.6	45.6
**S**	5.2	2.6
**Fe**	0.9	5.9
**Si**	4.4	9.9
**Al**	3.7	6.4

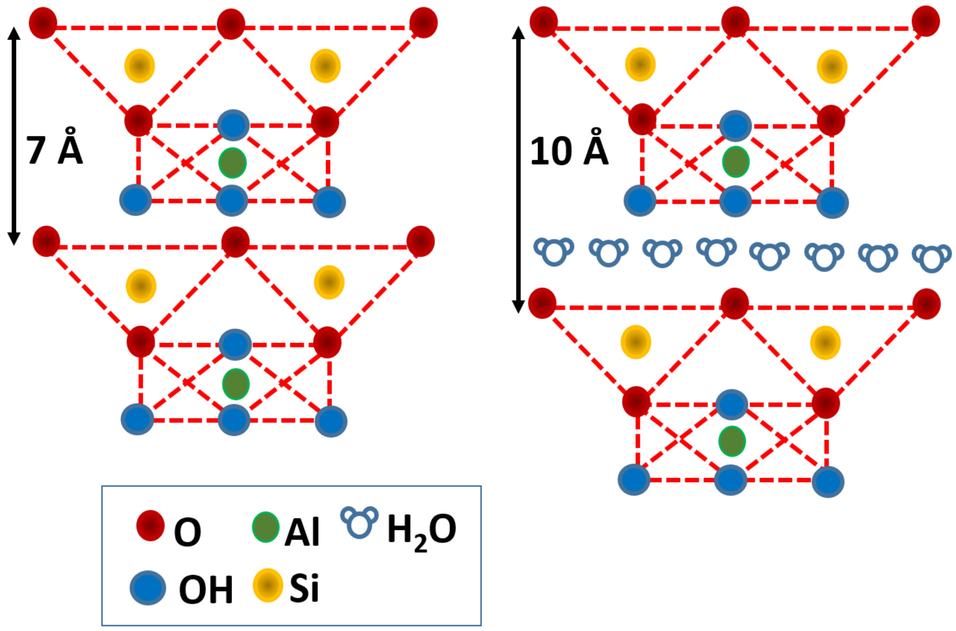

Figure S2. Scheme of clay crystal structures.

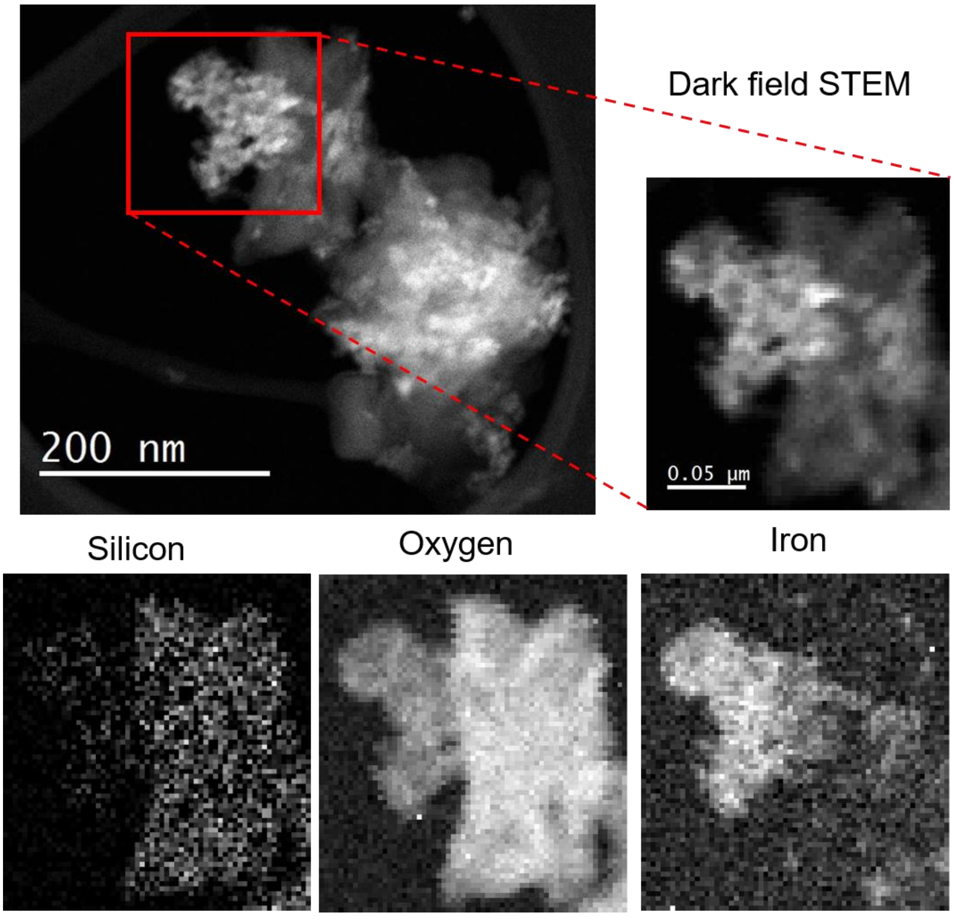

Figure S3. Dark field STEM images and chemical maps acquired on raw halloysite powder.

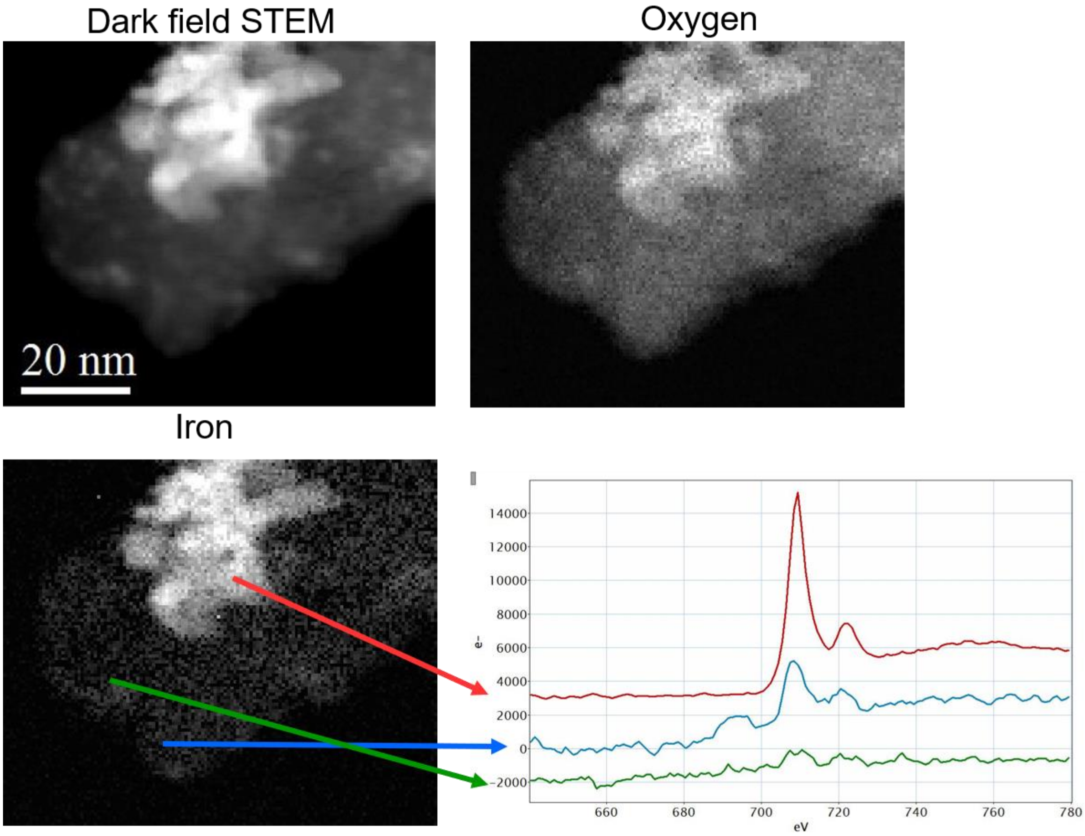

Figure S4. Chemical maps acquired on a platy structure and corresponding EELS spectra acquired on three different points.

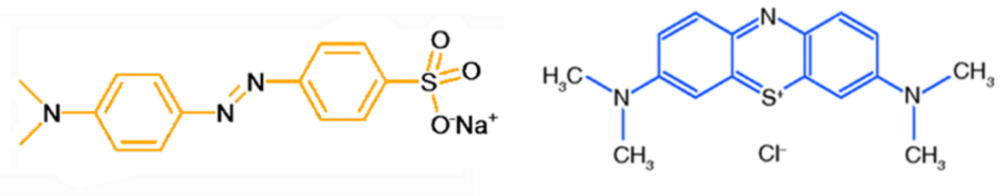

Figure S5. Chemical structures of Methyl Orange (on the left) and Methylene Blue (on the right) molecules.

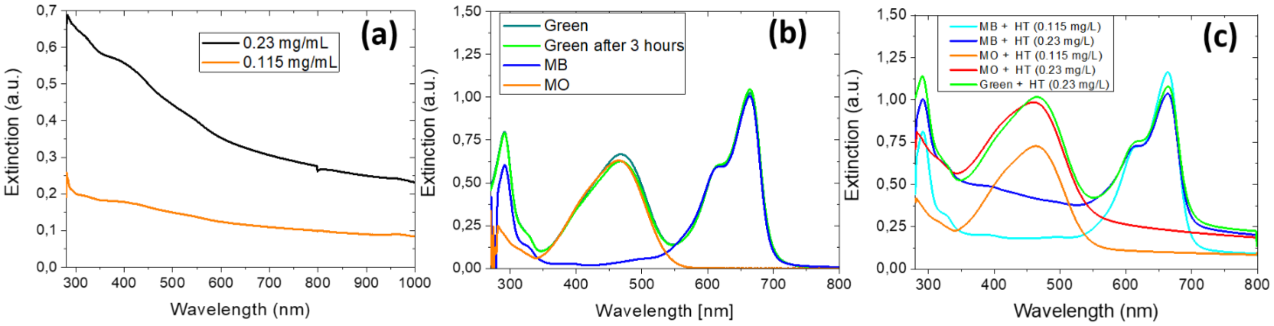

Figure S6. (**a**) UV-Visible spectra of raw halloysite powder dispersed in water at two different concentrations. (**b**) UV-Visible spectra of MO, MB and Green solutions (i.e. orange, blue and green curves, respectively). The spectrum for Green solution was acquired also after three hours. (**c**) UV-Visible spectra of MO, MB and Green solutions after few minutes of contact with halloysite powder at different concentrations.

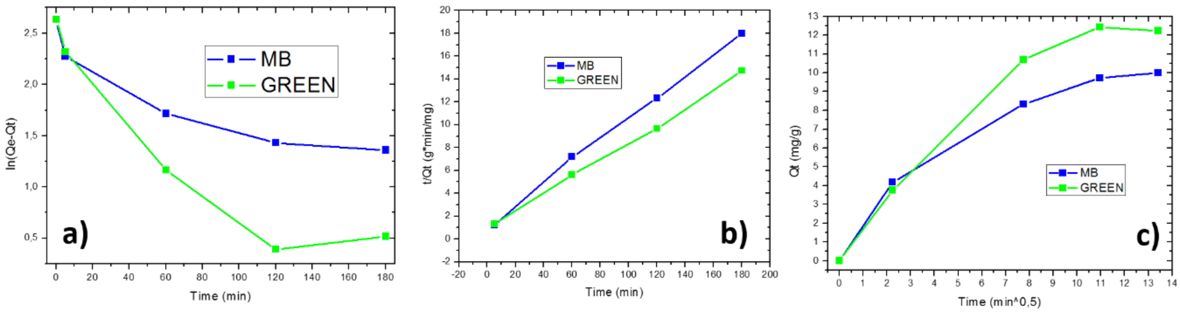

Figure S7. Linear expression of pseudo first order kinetic model (**a**), pseudo second order kinetic model (**b**) and intraparticle diffusion model (**c**) for MB adsorption in both MB and Green solutions (blue and green curves, respectively). It is clear, by a simple visual inspection, that only in case b) a reliable linear correlation of the experimental data is evidenced.

Table S2. R^2 values obtained by fitting the experimental data for MB removal for raw halloysite concentration of 0.23 mg/mL for MB and Green solutions, using the pseudo first order kinetic model, the pseudo-second-order kinetic model and the intraparticle diffusion model.

**Dye Solution**	R^2		
	Pseudo First Order	Pseudo Second Order $t/Q_t = (1/Q_e)t + (1/kQ_e^2)$	Intraparticle Diffusion Model $Q_t = k_d t^{0.5} + C$

	$\ln(Q_e - Q_t) = \ln Q_e - k_1 t$		
MB	0.92283	0.99672	0.9924
MB_Green	0.79499	0.99870	0.89693

Table S3. Amount of adsorbed dye on unit mass of clay at the equilibrium for increasing MB concentration both in single dye or mixed dyes solution.

Dye Solution	Q_e (mg/g) $[C_{MB}]_0$=3.2 mg/L	Q_e (mg/g) $[C_{MB}]_0$=6.4 mg/L	Q_e (mg/g) $[C_{MB}]_0$= 9.6 mg/L	Q_e (mg/g) $[C_{MB}]_0$= 12.8 mg/L
MB	8	15	21	24
MB_Green	10	20	31	34

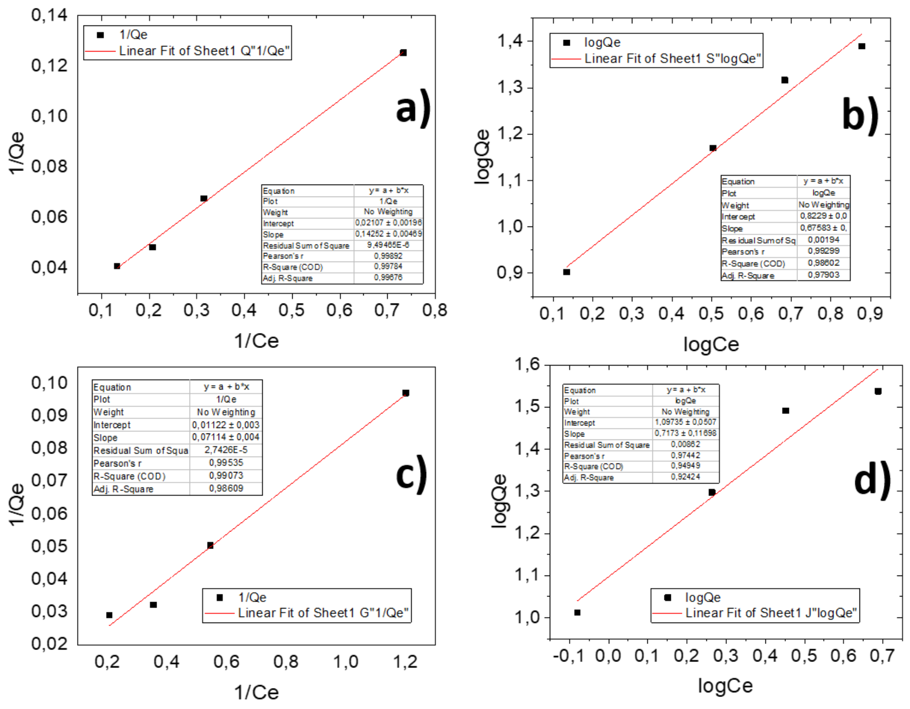

Figure S8. Langmuir (**a**,**c**) and Freundlich (**b**,**d**) plots for MB adsorption on Dunino clay in single (**a**,**b**) and mixed solution (**c**,**d**).

Table S4. R_L values obtained by Langmuir plots for MB adsorption in single and mixed dyes solution changing the MB initial concentrations.

**MB Concentration (mg/L)**	**R_L (Single Dye Solution)**	**R_L (Mixed Dye Solution)**
3.2	0.6646	0.6788
6.4	0.4977	0.5138
9.6	0.3978	0.4133
12.8	0.3313	0.3457
